# Clinical impact and cost-consequence analysis of ePlex® blood culture identification panels for the rapid diagnosis of bloodstream infections: a single-center randomized controlled trial

**DOI:** 10.1007/s10096-024-04820-z

**Published:** 2024-03-27

**Authors:** Yvan Caspar, A. Deves, C. Richarme, M. Le Marechal, L. Ponderand, A.-L. Mounayar, S. Lejeune, J. Arata-Bardet, M. Gallouche, C. Recule, D. Maubon, C. Garnaud, M. Cornet, M. Veloso, B. Chabani, M. Maurin, S. David-Tchouda, P. Pavese

**Affiliations:** 1 Laboratoire de Bactériologie-Hygiène Hospitalière, CHU Grenoble Alpes, Grenoble, France; 2 Univ. Grenoble Alpes, CNRS, CHU Grenoble Alpes, CEA, IBS, Grenoble, 38000 France; 3 Service des Maladies infectieuses et tropicales, CHU Grenoble Alpes, Grenoble, France; 4 Univ. Grenoble Alpes, CNRS, UMR 5525, VetAgro Sup, Grenoble INP, CHU Grenoble Alpes, TIMC, Grenoble, 38000 France; 5 Service d’Hygiène Hospitalière, CHU Grenoble Alpes, Grenoble, France; 6 Laboratoire de Parasitologie-Mycologie, CHU Grenoble Alpes, Grenoble, France; 7 University Grenoble Alpes, CNRS, CHU Grenoble Alpes, TIMC, Grenoble, 38000 France; 8 Cellule d’ingénierie des données, CHU Grenoble Alpes, Grenoble, France; 9 Unité d’évaluation médico-économique, Pôle Santé Publique, CHU Grenoble Alpes, Grenoble, France; 10 CIC 1406 Grenoble, INSERM, Grenoble, 38000 France; 11 Univ. Grenoble Alpes, TIMC-Imag UMR 5525, Grenoble, 38000 France

## Abstract

**Purpose:**

To assess clinical impact and perform cost-consequence analysis of the broadest multiplex PCR panels available for the rapid diagnosis of bloodstream infections (BSI).

**Methods:**

Single-center, randomized controlled trial conducted from June 2019 to February 2021 at a French University hospital with an institutional antimicrobial stewardship program. Primary endpoint was the percentage of patients with optimized antimicrobial treatment 12 h after transmission of positivity and Gram stain results from the first positive BC.

**Results:**

This percentage was significantly higher in the multiplex PCR (mPCR) group (90/105 = 85.7% %, CI95% [77.5 ; 91.8] vs. 68/107 = 63.6%, CI95% [53.7 ; 72.6]; *p* < 10^− 3^) at interim analysis, resulting in the early termination of the study after the inclusion of 309 patients. For patients not optimized at baseline, the median time to obtain an optimized therapy was much shorter in the mPCR group than in the control group (6.9 h, IQR [2.9; 17.8] vs. 26.4 h, IQR [3.4; 47.5]; *p* = 0.001). Early optimization of antibiotic therapy resulted in a non-statistically significant decrease in mortality from 12.4 to 8.8% (*p* = 0.306), with a trend towards a shorter median length of stay (18 vs. 20 days; *p* = 0.064) and a non-significant reduction in the average cost per patient of €3,065 (*p* = 0.15). mPCR identified all the bacteria present in 88% of the samples.

**Conclusion:**

Despite its higher laboratory cost, the use of multiplex PCR for BSI diagnosis leads to early-optimised therapy, seems cost-effective and could reduce mortality and length of stay. Their impact could probably be improved if implemented 24/7.

**Supplementary Information:**

The online version contains supplementary material available at 10.1007/s10096-024-04820-z.

## Introduction

Despite impressive technological revolutions in clinical microbiology over the last two decades, bloodstream infections (BSI) remain severe and have high mortality rates. In 2017, there were 11 million deaths due to BSI, accounting for 19.7% of global mortality [1]. Moreover, antimicrobial resistance (AMR) resulted in an estimated 1.27 million attributable deaths in 2019 and was responsible for 47.9 million disability-adjusted life-years, with BSI being the second infectious syndrome responsible for these high numbers [2]. The administration of antimicrobials within one hour of sepsis recognition is recommended by the Surviving Sepsis Campaign, as the prognosis of patients relies on the prompt initiation of effective antibiotic treatment [3]. Any delay in appropriate antibiotic therapy has recently been confirmed to gradually increase mortality between 12 h and 72 h after blood culture (BC) collection [4, 5]. However, the diversity of pathogens and increase in AMR reduce the efficacy of empirical antimicrobial treatment, which is neither effective nor optimal in 5 to 20% of BSI when evaluated at 48 h after BC collection [5, 6].

Several rapid molecular or MALDI-TOF-based techniques and rapid antimicrobial susceptibility testing (AST) methods have been developed for BSI. They shorten the time to identify the pathogen(s) and report initial data on their sensitivity to antibiotic treatment (i.e., the presence or absence of important antibiotic resistance genes or rapid antibiotic susceptibility testing results) [7–13]. However, many laboratories face difficulties in implementing these new methods on a 24/7 basis because of methodological hands-on time, limited service time, or high costs [8, 9, 14–17]. A survey of 209 laboratories from 25 European countries in 2017 showed that only 13% of laboratories started immediate processing of positive BC bottles 24/7, and that only 43.5% of laboratories performed both rapid identification and direct AST from positive BC [14].

While first-generation molecular panels only identified a limited number of microorganisms and resistance mechanisms, further developments have led to broader molecular panels that can detect more than 60 pathogens and resistance markers [7, 8, 10]. Their clinical and economic benefits have not been assessed in randomized controlled trials (RCTs) since the evaluation of the first generation FilmArray® Blood Culture Identification (BCID) panel, which identified only 24 bacterial or fungal species/genera and three resistance mechanisms [8]. In this study, we performed a single-center RCT to evaluate the clinical and economic impact of the broadest multiplex PCR panels available for BSI diagnosis in a French University hospital with an institutional antimicrobial stewardship program (ASP) and low resistance rates.

## Methods

### Study design

The HEMOFAST study (NCT03876990) was a single-blinded, single-center RCT conducted at Grenoble University Hospital (2,100 beds, including 64 ICU beds; approximately 100 000 inpatients per year). Eligible patients were adults suspected of sepsis with a detectable organism(s) growing in a positive BC confirmed by Gram stain (T0). Patients were enrolled by infectious diseases physicians from the ASP team only Monday to Friday during the laboratory opening hours (8:00 AM to 6:00 PM). After obtaining written consent, patients were randomized by the clinical microbiologist using the eCRF into two parallel groups (1:1 ratio, random bloc size 6,8,10 generated by the Data Stat team): the standard of care (SoC) group or the intervention group (mPCR), with the latter benefiting from multiplex PCR (mPCR) in addition to SoC (see supplementary material). Patients meeting at least one of the following criteria were not included: patients hospitalized in a palliative care unit or with an estimated survival before sepsis of less than one month, and persons referred to in articles L1121-5 to L1121-8 of the French Public Health Code (pregnant women, person deprived of liberty or unable to consent and children). This study was approved by the French ethics committee (IDRCB 2018-A02026). We used the CONSORT reporting guidelines [18].

### Laboratory testing

All positive BC were processed according to SoC procedures, including subculture, identification by MALDI-TOF mass spectrometry after overnight incubation (first digital imaging of the plates after 14 h of incubation) and AST (disk-diffusion method directly from positive BC with reading after 16–24 h incubation or broth microdilution from colonies) according to a previously described method with minor modifications [15]. In brief, blood cultures (BD Bactec Plus Aerobic/F, Lytic/10 Anaerobic/F, or Peds Plus/F; Becton Dickinson, Pont de Claix, France) were incubated in a BD Bactec™ Fx incubator. Overnight-positive BCs after 11 PM were handled the next morning during laboratory service time from 8:00 AM to 6:00 PM (Monday to Friday). BCs detected positive were confirmed by microscopic examination after Gram staining. An aliquot of positive blood culture was transferred into a dry tube (BD Vacutainer) and diluted to 1/50 (if the Gram stain showed Gram-positive cocci in clusters [GPCC] or Gram-negative rods) or to 1/5 (if the Gram stain showed Gram-positive cocci in pairs or chains [GPCP]) with saline solution according to the recommendations of CASFM-EUCAST for the direct disk-diffusion method from positive BC [19]. Then, bacterial suspension in saline solution were automatically subcultured by streaking nonselective and selective media using the automated BD Kiestra™ Work Cell Automation (WCA). Inoculated agar media were based on the Gram stain result (two 5% sheep blood Columbia or Polyvitex agar [one for aerobic and one for anaerobic incubation]) and, if required, based on the Gram stain (Drigalski medium, 5% sheep blood Columbia CNA agar or CAN2 medium for fungus [BioMérieux, Marcy l’Etoile, France]). Aerobic agar media were automatically incubated in connected incubators of the WCA (Drigalski and CAN2 under ambient atmosphere; Columbia blood agar and Polyvitex agar under a 5% CO2 enriched atmosphere). The anaerobic media were incubated in anaerobic jars using a gas pack (AnaeroGen, Oxoid). Digital imaging and reading of the agar plates at 14 h of incubation were performed using BD Kiestra™ WCA (extended to 24–48 h of incubation if required). Colonies were identified after a minimum of 14 h of growth using MALDI-TOF mass spectrometry (Microflex LT, Bruker Daltonics, Bremen, Germany). Antibiotic and antifungal susceptibility testing from colonies were performed using BD Phoenix™ panels PMIC-96 for *Staphylococcus* and *Enterococcus* strains and if required using NMIC-417 for GN bacilli. If required, discrimination between ESBL production and hyperexpression of the *ampC* gene was performed phenotypically using double disc synergy test and cloxacillin-containing Mueller-Hinton media, according to CASFM-EUCAST (Comité de l’Antibiogramme de la Société Française de Microbiologie – European Committee on Antimicrobial Susceptibility Testing) guidelines [19].

In the mPCR group, the appropriate GenMark Dx ePlex® BCID Panel (GenMark Diagnostics, a member of the Roche Group) was performed according to the Gram stain results of the first positive BC for each patient randomized in the mPCR group. mPCR was run as soon as possible and always within 12 h of positivity, as recommended by the manufacturer, by adding 50 µL of positive BC into the appropriate cartridge. The ePlex® BCID Panels identify 56 species or genera of bacteria and fungi, 3 Pan Targets (Pan Gram-Negative, Pan Gram-Positive, and Pan *Candida*) and 10 resistance genes in approximately 90 min (Figure S1). If a combination of GP and GN bacteria or a combination of bacteria and micromycetes was observed on the same or different blood culture bottles (or in case a Pan Gram-Negative, Pan Gram-Positive, or Pan *Candida* target was detected, although unsuspected), the complementary ePlex® BCID Panel(s) was tested. mPCR testing was performed only during working hours. PCR results were provided by phone to the ASP team and transmitted electronically to medical units.

### Antimicrobial stewardship program (ASP)

The ASP consists of a mobile infectious disease team that reviews all positive BC results in real time, moving through medical units to see patients if necessary, providing audits and feedback on management, treatment, and infection control to the medical units until the final microbiological results are obtained. Institutional ASP remained unchanged throughout the study period. The ASP team provided his advice daily as usual. However, final decision of treatment choice always remained to the physician in charge of the corresponding patient.

### Outcomes

The primary endpoint was the percentage of patients with optimized antimicrobial treatment 12 h (T12h) after transmission of positivity and Gram stain results from the first positive BC (T0). The T12h time point was relevant because it allowed for informed consent, randomization, mPCR testing, and antibiotic modification based on the mPCR results but not on any other SoC microbiological results that were obtained after at least 14 h of incubation. As investigators could not be blind, effective and optimal antibiotic therapy, source of infection or blood culture positivity due to contaminants were assessed during the weekly multidisciplinary meeting that is part of local ASP for bacteremia. These meetings included at least one infectious disease physician member of the ASP team, one clinical microbiologist and one infection control specialist but more may have been present at each meeting. The main outcome was assessed once, when all microbiological data were available (identification and AST results using standard of care method).

We defined effective and optimized treatment and contaminants as follows:

Effective treatment was defined as the first line of antibiotic treatment received by the patient which had an effective antibacterial activity against the pathogen(s) causing the BSI episode according to its AST profile. It could be the empiric antibiotic treatment started after suspicion of sepsis or after transmission of blood culture positivity and Gram stain result, if it was effective. Otherwise it was the first effective treatment after escalation of the treatment. However it could be not optimal according to best practice once AST data were available and source of infection identified because: 1/antibacterial spectrum was too large according to AST results and the source of infection identified (e.g. piperacillin/tazobactam for a pyelonephritis due to a 3GC-susceptible *E. coli*); 2/the treatment was associated to risk of selection of resistance (e.g. group-3 *Enterobacterales* such as *E. cloacae* complex treated with 3GC); 3/the treatment could be more toxic or associated to more side effects (e.g. *S. agalactiae* or methicillin-susceptible staphylococci bacteremia treated with vancomycin), 4/ the treatment had an suboptimal clinical efficacy because of limited diffusion in the primary site of infection….

Optimized antimicrobial treatment was defined as optimal intravenous antimicrobial treatment according to the species identified, final AST profile, and current French treatment guidelines depending on the source of infection (considering optimal clinical efficacy, potential side effects and selection of bacterial resistance but not dosing) (see supplementary material and Tables S1-S4 for extensive details about optimized treatment categorization in both groups and about reasons for suboptimal treatment at T12h). Optimized treatment corresponded to T0 treatment if already optimized, escalation of the antibiotic treatment, switching to a more effective compound or de-escalation (see supplementary material for definitions of antibiotic escalation, de-escalation and optimization). Most of the time, the antibiotic treatment considered as optimized treatment was the definitive intravenous treatment received by the patient for the bacteremia or fungemia episode, after adjustments of the antibiotic therapy based on the recommendation of the antibiotic stewardship team (Table S1), AST data and the primary source of infection identified. When intravenous treatment was not effective or not optimal but the first oral treatment was, the first oral treatment was considered optimized treatment. If the empirical treatment before T0 was considered optimized, the delay for the optimized treatment was considered to be 0. The assessors of primary endpoint were not blind of study group but participants to the multidisciplinary meeting had no information about the delays in setting up the different antibiotic treatments for each patient used for the calculation of the primary endpoint. Reasons for suboptimal treatment and categorization of patients with suboptimal treatment at T12h by the experts are described in Table S2.

Contaminants were defined as a single positive blood culture (or several from a single blood culture draw) for a given patient, showing one or several bacterial species that belong to potential skin commensals (e.g. coagulase-negative Staphylococci, *Corynebacterium* sp…) or known environmental contaminants (e.g.: *Micrococcus* sp), in the absence of any other site of infection with the same isolated organism and/or in the absence of local signs of infections if the blood culture was sampled from a catheter or a central line.

The secondary objective was a cost-consequence analysis (CCA) which is one of the tools used to carry out a cost-effectiveness evaluation. It analyses both the costs and health outcomes of one or more interventions from a broad perspective and reports them separately, to present to decision makers a summary of the different costs and effects. In this study, the economic evaluation compared the costs and clinical consequences for patients in both groups (initial stay with a 30-day endpoint) from the hospital’s perspective. Hospitalizations were evaluated using their corresponding case-mix costs from the latest available French National Cost Study (ENC, 2019). The cost of the intervention was estimated at €150 per patient. This cost includes the acquisition of the testing machine, labor cost, and reagent costs. We did not adjust the cost according to the competing effect of mortality in order to minimize the effect observed (conservative approach). The studied clinical consequences included delay in effective and optimized antibiotic therapy after T0, 30-day all-cause mortality, length of stay (LOS), and duration of treatment with the main broad-spectrum antibiotics. Post-hoc analyses described the percentage of effective and optimized treatments since T0, the time to results of microbiological data, and duration of broad-spectrum antibiotics restricted to four days after results of Gram stain.

### Statistical analysis

Assuming 40% and 55% of the optimized antibiotic therapy at T12h for patients in the SoC and mPCR groups, respectively [16], 173 subjects were required per group for a statistical power of 80% and a 5% two-sided alpha risk. Assuming 15% missing data or withdrawal of consent, 200 patients were to be included in each group. An interim analysis was scheduled after prolonging the study period owing to the COVID-19 pandemic, without changing the initial assumptions, in order to detect earlier a benefit of the innovative diagnostic approach and avoid a loss of chance for future patients in the standard arm. The interim analysis significance threshold was 0,003 for the primary outcome (O’Brien and Fleming method). In the context of the limited risk of the study, enrolment was pursued during the time required for interim analysis to enable the further use of innovation.

Quantitative variables are presented as mean and standard deviation (SD) or median and interquartile range [IQR] depending on the distribution of the data. Categorical variables were presented as numbers and percentages with 95% confidence intervals, if necessary. Missing data were not considered in the expression of percentages. Variables were compared using Student’s t-test or Mann-Whitney U test for continuous variables. Categorical variables and primary endpoints were compared using Chi squared test, after verification of the Cochran criteria, or Fisher’s exact test alternatively. The threshold for significance of the results was set at 0.05. For PCR performance compared to the SoC procedure, we calculated the positive and negative percent agreements (PPA and NPA) for each target using the numbers of true positives, false positives, true negatives, and false negatives (TP, FP, TN, and FN) as follows: PPA (%) = 100 × TP/(TP + FN) and NPA = 100 × TN/ (TN + FP).The analysis was performed using the STATA software (*StataCorp. 2017. Stata Statistical Software: Release 15. College Station, TX: StataCorp LLC)*.

## Results

### Patients

**Fig. 1:**
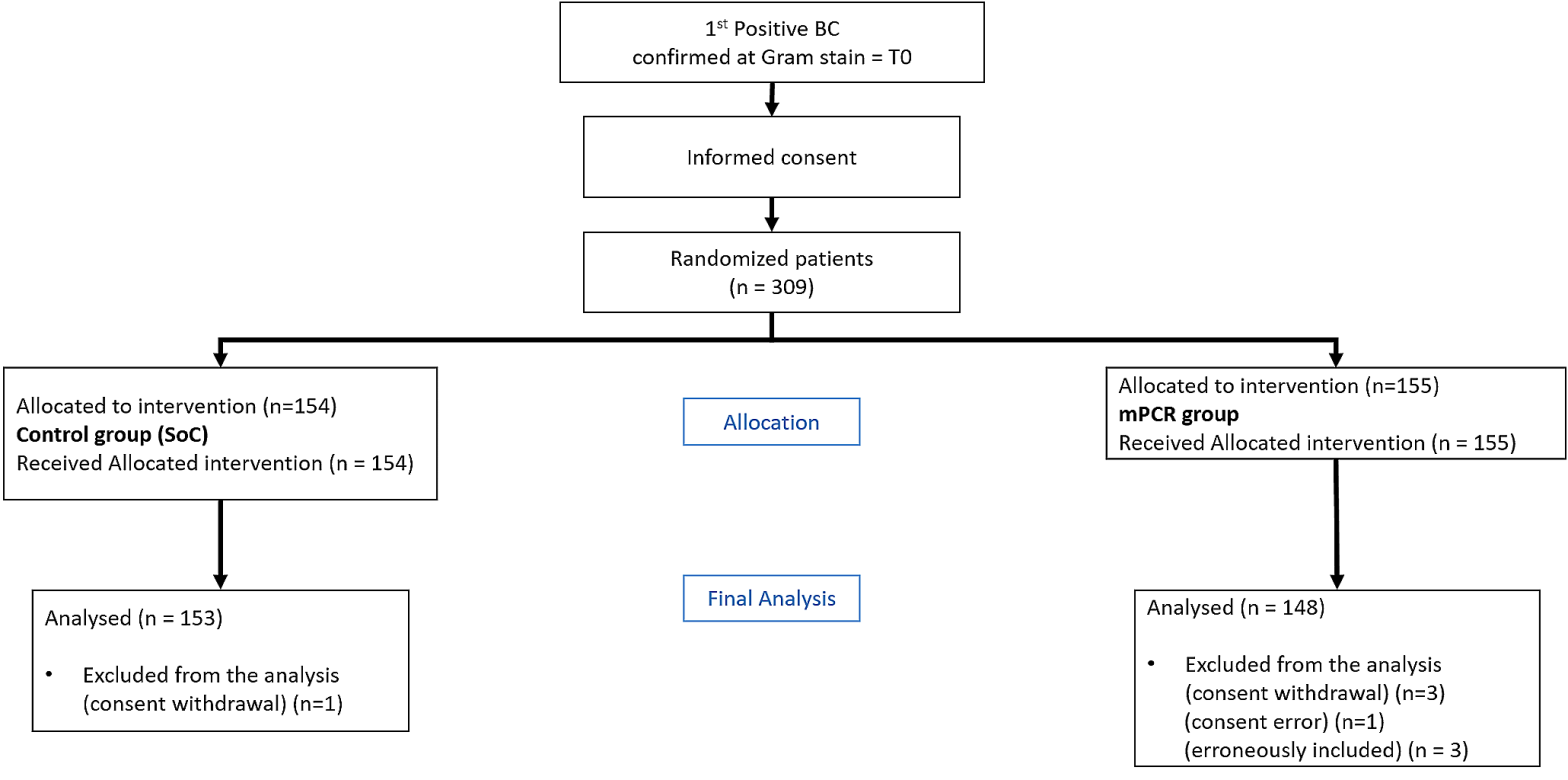
Flow diagram of the study

From June 2019 to February 2021, 309 patients could be enrolled, randomized and followed up for 30 days or until death (Fig. 1). The demographic and clinical characteristics are reported in Table 1.

**Table 1:** Demographic and clinical characteristics of patients

Characteristic	SoC group (n = 153)	mPCR group (n = 148)
Age, med [IQR]	68 [53 ; 75]	65 [55 ; 78.5]
Male sex	97 (63.4)	100 (67.6)
BMI, med [IQR]	23.9 [21.3 ; 28]	24.9 [21.9 ; 28.7]
Comorbidities:		
Cancer	74 (48.4)	51 (34.5)
Chronic cardiac disease	32 (20.9)	38 (25.7)
Chronic renal disease	24 (15.7)	25 (16.9)
Chronic pulmonary disease	18 (11.8)	17 (11.5)
Chronic liver disease	17 (11.1)	16 (10.8)
Solid organ transplant	5 (3.3)	6 (4.1)
Bone marrow transplant	13 (8.5)	12 (8.1)
HIV	0 (0)	3 (2)
Immunodeficiency:	60 (39.2)	42(28.4)
Chemotherapy within 90 days Immunosuppressive treatment Long-term corticosteroids	46 (76.7) 11 (18.3) 5 (8.3)	27 (64.3) 14 (33.3) 6 (14.3)
Creatinine (µmol/l) - med [IQR]	*n* = 147 73 [53 ; 116]	*n* = 143 85 [59 ; 116]
Leucocytes (G/l) - med [IQR]	*n* = 146 9.9 [4.7 ; 14.4]	*n* = 142 9.9 [5.5 ; 14.7]
CRP - med [IQR]	*n* = 117 98 [40 ; 208]	*n* = 115 100 [3 ; 168]
Medical ward at the time of BC collection		
- Emergency department	31 (20.3)	37 (25)
- Intensive care unit	29 (19)	21 (14.2)
- Hematology	21 (13.7)	21 (14.2)
- Other clinical ward	72 (47)	69 (46.6)
Medical ward at the time of 1st positive BC - Intensive care unit - Emergency department - Other clinical ward	42 (27.5) 21 (13.7) 90 (58.8)	30 (20.3) 24 (16.2) 94 (63.5)
Final diagnosis - Bacteremia/Fungemia - Contaminant	136 (88.9) 17 (11.1)	133 (89.9) 15 (10.1)
Source of BSI (Bacteremia/Fungemia) - Urinary - Digestive - Catheter - Pulmonary - Other	33 (24.3) 32 (23.5) 33 (24.3) 9 (6.6) 29 (21.3)	34 (25.6) 30 (22.6) 25 (18.8) 3 (2.3) 41 (30.8)

*Abbreviations* SoC, standard of care; mPCR: multiplex polymerase chain reaction; IQR, interquartile range

Data are presented as Numbers (%) unless otherwise specified

9 and 10 missing values of BMI were observed in the SoC and mPCR groups, respectively

### Primary outcome

In the interim analysis, the percentage of patients with an optimized treatment 12 h after validation of first BC positivity and Gram stain result was significantly superior in the mPCR group (90/105 = 85.7%, CI95% [77.5 ; 91.8] vs. 68/107 = 63.6%, CI95% [53.7 ; 72.6]; *p* < 10^− 3^), resulting in the early termination of the study when the outcomes were delivered. The final analysis confirmed previous results: 122/148 (82.4%, CI95% [75.3% ; 88.2%]) vs. 93/153 (60.8%, CI95% [52.6% ; 68.6], *p* < 10^− 3^).

### Cost-consequence analysis and secondary clinical outcomes

**Fig. 2:**
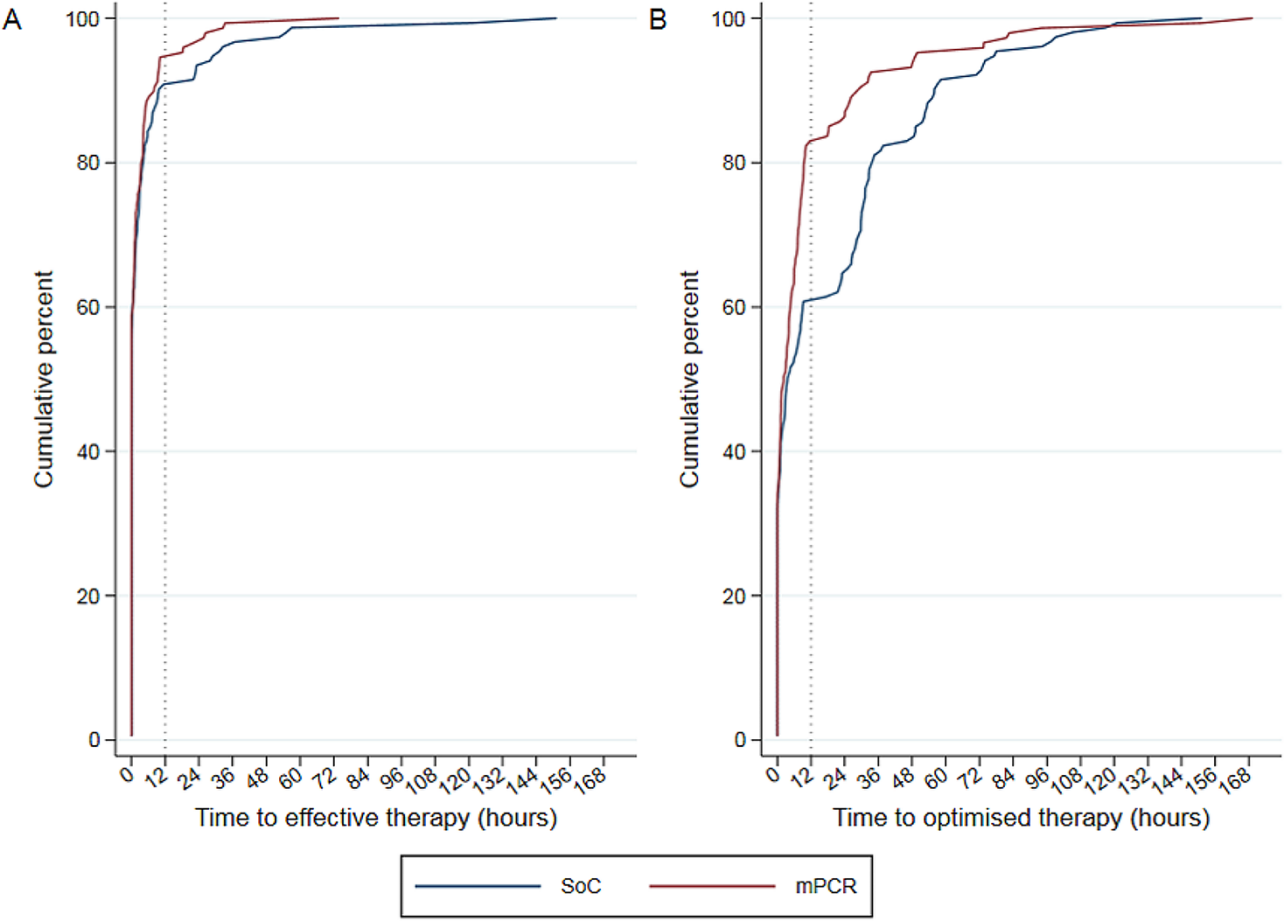
Cumulative percentage of effective (**A**) and optimized (**B**) antibiotic therapy rates after transmission of results of the Gram stain from the first positive BC (T0). *p* < 10^− 3^for the primary outcome (percentage of patients with an optimized treatment 12 h after validation of first BC positivity and Gram stain result in both groups) (chi-square test)

Main secondary outcomes are presented in Table 2. The 30-day mortality rate was lower in the mPCR group (8.8 vs. 12.4%); however, this difference was not statistically significant (*p* = 0.306). A trend towards a shorter median LOS was observed in the mPCR group (18 days vs. 20 days; *p* = 0.064). The rates of effective or optimized treatment at T0 did not differ between the mPCR and SoC groups (58.8% vs. 56.2% [*p* = 0.651] and 33.8% vs. 32% [*p* = 0.746], respectively) (Fig. 2). The median time to effective treatment did not differ between the groups, even for patients who did not receive any effective antibiotic therapy at baseline. The rates of effective treatment at T12h were 94.6 vs. 90.8% (*p* = 0.21) in the mPCR and SoC groups, respectively. However, in patients who did not receive optimized therapy at T0, the median time to obtain an optimized therapy was much shorter in the mPCR group than in the control group (6.9 h, IQR [2.9; 17.8] vs. 26.4 h, IQR [3.4; 47.5]; *p* = 0.001).

**Table 2:** Cost-consequence comparison

	SoC group (n = 153)	mPCR group (n = 148)	p-value
Health Outcomes			
30-day all-cause mortality, n (%)	19 (12.4%)	13 (8.8%)	0.306
Median length of stay^#^	20 days [10;36]	18 days [7;29]	0.064
Median time to effective treatment^#^			
-For all patients	0 h [0;2.8]	0 h [0;2.2]	0.536
-For patients not receiving effective treatment at T0	(*n* = 67)	(*n* = 61)	
	3.5 h [1.4;9.4]	3.4 h [1.3;7.9]	0.537
Median time to optimized treatment^#^			
-For all patients	3.7 h [0;31.3]	2.2 h [0;8.6]	0.026
-For patients not optimized at T0	(*n* = 104)	(*n* = 98)	
	26.4 h [3.4 ; 47.5]	6.9 h [2.9 ; 17.8]	0.001
Costs			
Mean cost of the initial hospital stay (SD)*	19,973€ (19,785€)	16,758€ (17,351 €)	
mPCR costs**		150€	
TOTAL costs	19,973€ (19,785€)	16,908€ (17,351 €)	0.1543^1^

*Abbreviations* SoC, standard of care; mPCR: multiplex polymerase chain reaction; IQR, interquartile range, SD, standard deviation. Median times are presented with [IQR]

^#^After T0 (transmission of BC and Gram stain result)

^1^T-test

*Hospital data were taken from the French 2019 national hospital common cost study. Only 147 patients in the mPCR group for cost analysis. One patient was excluded from the analysis due to lack of data on his initial hospitalization

**assumption taking into account the cost of mPCR cartridges and of the automaton (financial amortization over 7 years) and the cost of staff to run the analysis and report results

The difference in the average cost per patient was €3,065, SD [-1,159 €, 7 289 €], but this trend was not significant (*p* = 0.15) (Table 2). The consumption of broad-spectrum antibiotics did not significantly differ between the two groups when limited to 96 h after Gram stain and was therefore not valued (Table S5).

### Microbiological data and performance of mPCR

Microbial diversity and prevalence of resistance markers were comparable between the two groups (Tables S6-S8). Among the positive BCs in the mPCR group (Tables S9-S12), 132/148 (89.2%) were monomicrobial (58 GP, 71GN, and 3 yeast) and 16 /148 (10.8%) polymicrobial. In the SoC group, 136 BSI were monomicrobial (55 GP, 76GN, and 5 yeast) and 17 (11.1%) were polymicrobial. Gram stain detected less than 5% of the samples with yeast or polymicrobial morphologies (Table S6). A correct identification at the genus or species level, compared with SoC, was obtained for 119/132 (90.2%) of monomicrobial BC, increasing to 119/126 (94.4%) after exclusion of off-panel targets (Supplementary material Table S9-S11). All bacteria were detected in 11/16 (69%) polymicrobial samples, and this number increased to 11/13 (85%) after the exclusion of samples with off-panel bacteria (Table S12). No false-positive results were observed for any target but five false-negative results were obtained (Table S13). *S. epidermidis* was not detected in three samples (two polymicrobial), and one each of *E. coli* and *C. freundii* were not detected but have all been considered as contaminants when SoC results were available.

Regarding resistance targets, the *mecA* gene was detected in 16/17 (94%) samples with methicillin-resistant Staphylococci (15/24 samples with CoNS and in 1/14 *S. aureus* strains) and the *bla*_*CTX−M*_ gene was detected in 4/6 (67%) samples with extended spectrum beta-lactamase (ESBL)-producing Gram-negative bacteria (GNB) (one invalid cartridge; one undetected target). No false-positive results were observed for any target. One ESBL-producing *E. cloacae* strain was not detected because of an invalid cartridge, the *bla*_*CTX−M*_ gene was not detected in an *E. coli* strain, and a *mecA-*positive *S. epidermidis* strain could not be detected in one sample because the *S. epidermidis* target was also not detected. Overall, the rate of invalid cartridges was 19/155 (12.2%) after the first run (10 consumable error, 5 instrument errors, 3 possible technical error with overloading sample into the consumable, 1 unknown) and 4/155 (2.6%) after retesting. Seven discrepancies (4.7%) were identified (Table S13).

**Fig. 3:**
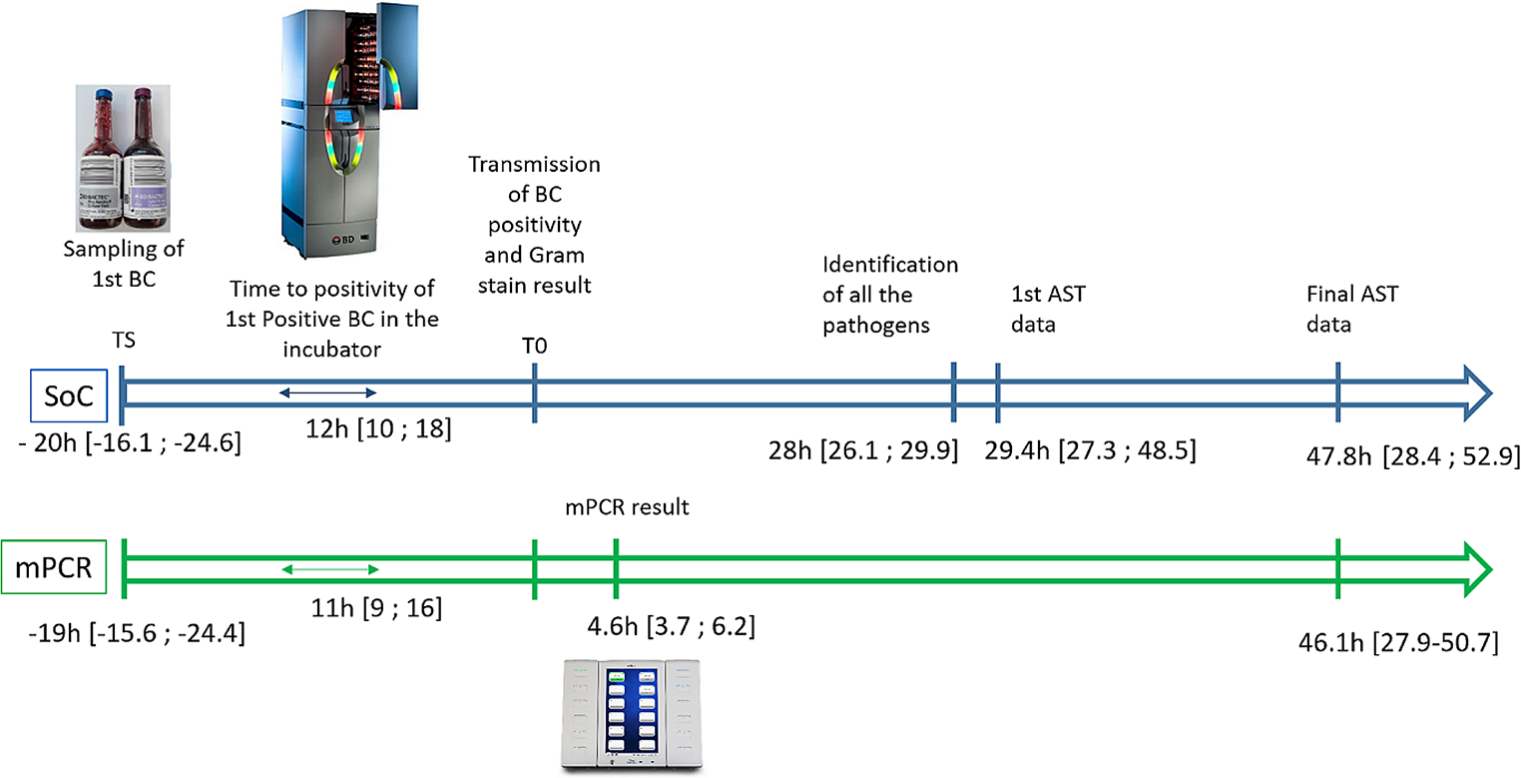
Median time to results of microbiological data in the mPCR and SoC groups. *Abbreviations* SoC: standard of care; mPCR: multiplex polymerase chain reaction; BC : blood culture; AST: antimicrobial susceptibility testing; TS: time of sampling of first BC for the sepsis episode; T0: transmission of 1st positive BC positivity and Gram stain results. All data are expressed as median [IQR]. 1st AST data corresponds to the first transmission of antibiotic susceptibility results. Final AST data corresponded to the final validation of full antibiotic susceptibility testing data that could require automated AST from isolated colonies in case of polymicrobial samples, if growth was insufficient using disk diffusion from positive blood culture or if a Staphylococcus species was identified in particular to have AST results for glycopeptides and daptomycin that could not be obtained by disk diffusion method

Time to results (TTR) of different laboratory tests in both groups is shown in Fig. 3. Median TTR for mPCR after Gram stain result was 4.6 h, IQR [3.7–6.2] corresponding to a median TTR of 24.1 h, IQR [20.2–30.2] from the sampling of the first BC. It was significantly reduced compared to the SoC median time to identification of all pathogens (28 h, IQR [26.1–29.9]; *p* < 10^− 3^) and to obtain the first AST data (29.4 h, IQR [27.3; 48.8]; *p* < 10^− 3^).

## Discussion

In this trial, the use of mPCR in addition to SoC diagnostic procedures and ASP showed a 22% increase of the percentage of patients with an optimized treatment 12 h after the transmission of the Gram stain result of the first positive BC: 82.4% vs. 60.8% (*p* < 10^− 3^). While Gram stain results allowed to increase effective antibiotic treatment from 56 to 91%, 40% of antibiotic treatments were not optimal at that time. For those patients, the median time to obtain an optimized therapy was 19.5 h shorter in the mPCR group. Faster mPCR results were associated with a non-significant decrease in mortality, with a trend towards a shorter median LOS of two days and a reduced but non-significant average cost per patient of €3,065.

Over the last decade, several rapid phenotypic and genotypic methods for BSI diagnosis have been shown to reduce mortality and the time to effective or optimal therapy when an ASP is associated with the transmission of results [7–10, 12, 13, 20–24]. However, conclusions regarding mortality, time to discharge, and time to appropriate antibiotic treatment were not significant in a recent meta-analysis [25]. Few studies have been prospective trials, and none have evaluated the clinical impact of recent versions of broad mPCR panels (ePlex® BCID, Verigene® BCID, or FilmArray® BCID2 panels), especially in countries with low prevalence of AMR [8–10, 20].

Our data confirm the medical benefit that mPCR has on accelerating the time to results and improving antibiotic stewardship for a large number of patients, even in hospitals with long-standing ASP and low resistance rates. These data are in agreement with previous results which showed acceleration of time to identification and/or faster changes in antibiotic therapy using mPCR, MALDI-TOF, or automated fluorescence in situ hybridization technology assays [8, 9, 21–23]. Twelve hours after the transmission of BC positivity, the first antibiotic adaptation based on Gram stain results allowed an increase in the percentage of patients with effective antibiotic treatment from 56 to 91%, but only 60% of antibiotic treatments were optimized at that time. mPCR stewarded more than 94% effective treatment at T12h and more than 82% optimized treatment.

Our data point towards a global trend in the clinical and economic benefits of the mPCR strategy, even though several secondary outcomes were not significant. This may be due to the fact that this trial was not powered to detect differences, as previously experienced, and because of the early termination of the study [8, 9, 21]. We observed a trend for the reduction of the use of some broad-spectrum antibiotics (piperacillin/tazobactam and daptomycin), while the use of third-generation cephalosporins (3GC) was increased, but these trends were not significant (Table S5) compared to previous studies using mPCR assays for rapid diagnosis of BSI [8].

Overall, the ePlex® BCID mPCR was able to identify all bacteria present in 88% of the BC samples, including 69% of the polymicrobial samples for which MALDI-TOF-based rapid techniques are not reliable [15, 26–31]. In comparison, respectively 78% and 77% were identified in RCTs testing FilmArray® BCID or Accelerate PhenoTest® BC kit, and 89% in a prospective controlled clinical trial using rapid MALDI-TOF identification on bacterial pellets [8, 9, 21]. The median time to mPCR results from the sampling of the first BC for suspicion of sepsis was 24.1 h, which is lower than the mean time of 33 h observed in the latter trial using rapid MALDI-TOF identification of bacterial pellets that are usually performed in batches during opening hours [9]. Moreover, the Pan targets allowed the detection of three cases of polymicrobial bacteremia undetected by Gram stain: a *Bacteroides fragilis* strain associated with a *Streptococcus anginosus* group that was missed, an *S. mitis* strain associated with *E. coli*, and the presence of staphylococci in a sample positive for *E. cloacae*. Concordance between the resistance markers and SoC was observed in 98% of the samples. Quick detection of the presence or absence of resistance genes is of primary importance for early adaptation of antibiotic treatment, especially for methicillin-resistant staphylococci [20]. In contrast, though other resistance mechanisms to 3GC may be present in the absence of *bla*_*CTX−M*_ gene, mPCR helped in antibiotic escalation for Gram-negative BSI rather than de-escalation. The three episodes of fungemia were also correctly identified in line with previous studies [27, 32] The high mortality of fungemia reinforces the need for a rapid diagnostic technique. The longer growth period of micromycetes compared to that of bacteria delays the identification and determination of their susceptibility to antifungal drugs [33]. Identification of *Candida* species allows for minimal adaptation to antifungal therapy, particularly for species that are not very sensitive to fluconazole [34].

Our study had several limitations. First, according to French regulations, no waiver of consent was possible, delaying the mPCR result, which may have limited the expected benefits. The use of this assay 24/7 would also reduce the delay in antibiotic optimization and may improve other outcomes. Not all eligible patients could be enrolled during the study period. However, the pathogen diversity in this study was similar to that of the annual BSI epidemiology of our hospital. Our RCT was single-center, and thus may not be generalizable to all other hospital settings and countries. If higher resistance rates are present, the impact of rapid mPCR could be greater. We did not study the effect of this rapid test without an ASP. We did not use rapid identification with MALDI-TOF on bacterial pellets or short subcultures in the control group because this method was not available in our laboratory at the time of the study. Finally, our data also show that many other aspects of the “microbiologistics” of BSI still need improvement to reduce the delay in optimized treatment (e.g., reducing transport delays or 24/7 management of positive BC) [7].

In conclusion, this mPCR, which requires less than one hour of training, less than two minutes of hands-on time, and provides results in 90 min, has proven its efficacy in accelerating the optimization of antibiotic treatment in BSI. Despite its higher laboratory costs compared with the SoC strategy, the use of mPCR for BSI diagnosis appears cost-effective at short-term and might save money in the healthcare system. This assay can be easily performed 24/7 times by non-expert personnel. It may be used as a stand-alone automaton in microbiology laboratories or point-of-care or implemented along with other rapid diagnostic techniques, such as MALDI-TOF rapid identification, which is more difficult to run 24/7, or recent rapid AST methods [7, 21, 35]. The results must be associated with an effective ASP for optimal performance [8, 21].

### Electronic supplementary material

Below is the link to the electronic supplementary material.

Supplementary Material 1

## Data Availability

The protocol of the study and the datasets generated during the study are not publicly available but are available from the corresponding author on reasonable request.

## References

[1] Rudd KE, Johnson SC, Agesa KM, Shackelford KA, Tsoi D, Kievlan DR et al (2020) Global, regional, and national sepsis incidence and mortality, 1990–2017: analysis for the global burden of Disease Study. Lancet 395:200–211. 10.1016/S0140-6736(19)32989-7 31954465 10.1016/S0140-6736(19)32989-7 PMC6970225

[2] Murray CJ, Ikuta KS, Sharara F, Swetschinski L, Aguilar GR, Gray A et al Global burden of bacterial antimicrobial resistance in 2019: a systematic analysis. Lancet 2022 12;399:629–655. 10.1016/S0140-6736(21)02724-0 10.1016/S0140-6736(21)02724-0 PMC8841637 35065702

[3] Seymour CW, Gesten F, Prescott HC, Friedrich ME, Iwashyna TJ, Phillips GS et al Time to treatment and mortality during mandated Emergency Care for Sepsis. N Engl J Med 2017 8;376:2235–2244. 10.1056/NEJMoa1703058 10.1056/NEJMoa1703058 PMC5538258 28528569

[4] Evans L, Rhodes A, Alhazzani W, Antonelli M, Coopersmith CM, French C et al (2021) Surviving sepsis campaign: International guidelines for management of Sepsis and Septic Shock 2021. Crit Care Med 49:e1063–e1143. 10.1097/CCM.0000000000005337 34605781 10.1097/CCM.0000000000005337

[5] Van Heuverswyn J, Valik JK, van der Desirée S, Hedberg P, Giske C, Nauclér P association between time to appropriate antimicrobial treatment and 30-day mortality in patients with bloodstream infections: a retrospective cohort study. Clinical infectious diseases 2022 6;ciac727. 10.1093/cid/ciac727 10.1093/cid/ciac727 PMC9907509 36065752

[6] Robineau O, Robert J, Rabaud C, Bedos J-P, Varon E, Péan Y et al Management and outcome of bloodstream infections: a prospective survey in 121 French hospitals (SPA-BACT survey). IDR 2018;Volume 11:1359–68. 10.2147/IDR.S165877 10.2147/IDR.S165877 PMC6124465 30214256

[7] Lamy B, Sundqvist M, Idelevich EA, ESCMID Study Group for Bloodstream Infections (2020) Endocarditis and Sepsis (ESGBIES). Bloodstream infections - standard and progress in pathogen diagnostics. Clin Microbiol Infect 26:142–150. 10.1016/j.cmi.2019.11.017 31760113 10.1016/j.cmi.2019.11.017

[8] Banerjee R, Teng CB, Cunningham SA, Ihde SM, Steckelberg JM, Moriarty JP et al (2015) Randomized Trial of Rapid Multiplex polymerase chain reaction–based blood culture identification and susceptibility testing. Clin Infect Dis 161:1071–1080. 10.1093/cid/civ447 10.1093/cid/civ447 PMC4560903 26197846

[9] Osthoff M, Gürtler N, Bassetti S, Balestra G, Marsch S, Pargger H et al (2017) Impact of MALDI-TOF-MS-based identification directly from positive blood cultures on patient management: a controlled clinical trial. Clin Microbiol Infect 23:78–85. 10.1016/j.cmi.2016.08.009 27569710 10.1016/j.cmi.2016.08.009

[10] Timbrook TT, Morton JB, McConeghy KW, Caffrey AR, Mylonakis E, LaPlante KL (2017) The Effect of Molecular Rapid Diagnostic Testing on Clinical outcomes in Bloodstream infections: a systematic review and Meta-analysis. Clin Infect Dis 164:15–23. 10.1093/cid/ciw649 10.1093/cid/ciw649 27678085

[11] Jasuja JK, Zimmermann S, Burckhardt I (2020) Evaluation of EUCAST rapid antimicrobial susceptibility testing (RAST) for positive blood cultures in clinical practice using a total lab automation. Eur J Clin Microbiol Infect Dis 39:1305–1313. 10.1007/s10096-020-03846-3 32112163 10.1007/s10096-020-03846-3 PMC7303068

[12] Pliakos EE, Andreatos N, Shehadeh F, Ziakas PD, Mylonakis E The cost-effectiveness of Rapid Diagnostic Testing for the diagnosis of Bloodstream infections with or without Antimicrobial Stewardship. Clin Microbiol Reviews 2018 30;31:e00095–e00017, /cmr/31/3/e00095-17.atom.10.1128/CMR.00095-17 10.1128/CMR.00095-17 PMC6056844 29848775

[13] Zacharioudakis IM, Zervou FN, Shehadeh F, Mylonakis E Cost-effectiveness of molecular diagnostic assays for the therapy of severe sepsis and septic shock in the emergency department. PLoS ONE 2019 24;14:e0217508. 10.1371/journal.pone.0217508 10.1371/journal.pone.0217508 PMC6534337 31125382

[14] Idelevich EA, Seifert H, Sundqvist M, Scudeller L, Amit S, Balode A et al (2019) Microbiological diagnostics of bloodstream infections in Europe—an ESGBIES survey. Clin Microbiol Infect 25:1399–1407. 10.1016/j.cmi.2019.03.024 30980927 10.1016/j.cmi.2019.03.024

[15] Bryant S, Almahmoud I, Pierre I, Bardet J, Touati S, Maubon D et al (2020) Evaluation of Microbiological Performance and the potential clinical impact of the ePlex® Blood Culture Identification panels for the Rapid Diagnosis of Bacteremia and Fungemia. Front Cell Infect Microbiol 26:10:594951. 10.3389/fcimb.2020.594951 10.3389/fcimb.2020.594951 PMC7726344 33324578

[16] Timsit J-F, Soubirou J-F, Voiriot G, Chemam S, Neuville M, Mourvillier B et al (2014) Treatment of bloodstream infections in ICUs. BMC Infect Dis 14:489. 10.1186/1471-2334-14-489 25431091 10.1186/1471-2334-14-489 PMC4289315

[17] Kumar A, Roberts D, Wood KE, Light B, Parrillo JE, Sharma S et al Duration of hypotension before initiation of effective antimicrobial therapy is the critical determinant of survival in human septic shock*. Crit Care Med 2006 1;34:1589–1596. 10.1097/01.CCM.0000217961.75225.E9 10.1097/01.CCM.0000217961.75225.E9 16625125

[18] Schulz KF, Altman DG, Moher D, for the CONSORT Group. CONSORT 2010 Statement: updated guidelines for reporting parallel group randomised trials 10.3736/jcim20100702 20619135

[19] Société fraçaise de microbiologie CASFM / EUCAST V2.0 Mai 2019 [Internet]. doi: https://www.sfm-microbiologie.org/wp-content/uploads/2019/05/CASFM2019_V2.0_MAI.pdf

[20] Emonet S, Charles PG, Harbarth S, Stewardson AJ, Renzi G, Uckay I et al Rapid molecular determination of methicillin resistance in staphylococcal bacteraemia improves early targeted antibiotic prescribing: a randomized clinical trial. Clin Microbiol Infect 2016 1;22:946.e9-946.e15. 10.1016/j.cmi.2016.07.02210.1016/j.cmi.2016.07.022 27475737

[21] Banerjee R, Komarow L, Virk A, Rajapakse N, Schuetz AN, Dylla B et al Randomized Trial evaluating clinical impact of RAPid IDentification and susceptibility testing for Gram-negative bacteremia: RAPIDS-GN. Clin Infect Dis 2020 6;73:e39–46. 10.1093/cid/ciaa528 10.1093/cid/ciaa528 PMC8246790 32374822

[22] Ehren K, Meißner A, Jazmati N, Wille J, Jung N, Vehreschild JJ et al Clinical impact of Rapid species Identification from positive blood cultures with same-day phenotypic Antimicrobial susceptibility testing on the management and outcome of Bloodstream infections. Clin Infect Dis 2020 17;70:1285–1293. 10.1093/cid/ciz406 10.1093/cid/ciz406 31094414

[23] MacVane SH, Nolte FS Benefits of adding a Rapid PCR-Based blood culture identification panel to an established antimicrobial stewardship program. J Clin Microbiol 2016 1;54:2455–2463. 10.1128/JCM.00996-16 10.1128/JCM.00996-16 PMC5035429 27487951

[24] Venkatesh B, Schlapbach L, Mason D, Wilks K, Seaton R, Lister P et al Impact of 1-hour and 3-hour sepsis time bundles on patient outcomes and antimicrobial use: a before and after cohort study. The Lancet Regional Health – Western Pacific [Internet] 2022 1 [cited 2022 25];18. https://www.thelancet.com/journals/lanwpc/article/PIIS2666-6065(10.1016/j.lanwpc.2021.100305 10.1016/j.lanwpc.2021.100305 PMC8654968 35024649

[25] Anton-Vazquez V, Hine P, Krishna S, Chaplin M, Planche T Rapid versus standard antimicrobial susceptibility testing to guide treatment of bloodstream infection. Cochrane Database Syst Rev [Internet] 2021 4 [cited 2022 2];2021. https://www.ncbi.nlm.nih.gov/pmc/articles/PMC8561756/doi: 10.1002/14651858.CD013235.pub2 10.1002/14651858.CD013235.pub2 PMC8561756 34097767

[26] Huang T-D, Melnik E, Bogaerts P, Evrard S, Glupczynski Y (2018) Evaluation of the ePlex Blood Culture Identification panels for detection of pathogens in Bloodstream infections. J Clin Microbiol 28:57:e01597–e01518. /jcm/57/2/JCM.01597-18.atom 10.1128/JCM.01597-18 PMC6355516 30487304

[27] Oberhettinger P, Zieger J, Autenrieth I, Marschal M, Peter S (2020) Evaluation of two rapid molecular test systems to establish an algorithm for fast identification of bacterial pathogens from positive blood cultures. Eur J Clin Microbiol Infect Dis 4. 10.1007/s10096-020-03828-5 10.1007/s10096-020-03828-5 PMC7225181 32020397

[28] Carroll KC, Reid JL, Thornberg A, Whitfield NN, Trainor D, Lewis S et al Clinical performance of the Novel GenMark Dx ePlex® Blood Culture ID Gram-positive panel. J Clin Microbiol 2020 29;JCM.01730-19, jcm;JCM.01730-19v1. 10.1128/JCM.01730-19 10.1128/JCM.01730-19 PMC7098771 31996444

[29] Wolk DM, Young S, Whitfield NN, Reid JL, Thornberg A, Carroll KC et al A Multicenter Clinical Study to demonstrate the diagnostic accuracy of the GenMark Dx ePlex Blood Culture Identification Gram-negative panel. J Clin Microbiol [Internet] 2021 7 [cited 2022 15]. https://journals.asm.org/doi/abs/10.1128/JCM.02484-20 10.1128/JCM.02484-20 PMC8373019 34232066

[30] Tansarli GS, Chapin KC A closer look at the laboratory impact of utilizing eplex blood culture identification panels: a workflow analysis using rapid molecular detection for positive blood cultures. Microbiology Spectrum [Internet] 2022 7 [cited 2022 1]. 10.1128/spectrum.01796-22. https://doi.org/journals-asm-org.insb.bib.cnrs.fr/doi/10.1128/spectrum.01796-22. 10.1128/spectrum.01796-22 PMC9602361 36069598

[31] McCarty TP, White CM, Meeder J, Moates D, Pierce HM, Edwards WS et al Analytical performance and potential clinical utility of the GenMark Dx ePlex® blood culture identification gram-positive panel. Diagn Microbiol Infect Disease 2022 1;104:115762. 10.1016/j.diagmicrobio.2022.115762 10.1016/j.diagmicrobio.2022.115762 35988351

[32] Zhang SX, Carroll KC, Lewis S, Totten M, Mead P, Samuel L et al Multi-center evaluation of a PCR-based Digital Microfluidics and Electrochemical Detection System for the Rapid Identification of 15 fungal pathogens directly from positive blood cultures. J Clin Microbiol 2020 19;JCM.02096-19, jcm;JCM.02096-19v1. 10.1128/JCM.02096-19 10.1128/JCM.02096-19 PMC7180249 32075904

[33] Ransom EM, Alipour Z, Wallace MA, Burnham C-AD Evaluation of optimal blood culture incubation time to maximize clinically relevant results from a contemporary blood Culture Instrument and Media System. J Clin Microbiol 2021 18;59:e02459–e02420. 10.1128/JCM.02459-20 10.1128/JCM.02459-20 PMC8106720 33239377

[34] Maubon D, Dard C, Garnaud C, Cornet M (2018) Profile of GenMark’s ePlex® blood culture identification fungal pathogen panel. Expert Rev Mol Diagn 18:119–132. 10.1080/14737159.2018.1420476 29284316 10.1080/14737159.2018.1420476

[35] Kim J-H, Kim I, Kang CK, Jun K-I, Yoo SH, Chun JY et al Enhanced antimicrobial stewardship based on rapid phenotypic antimicrobial susceptibility testing for bacteraemia in patients with haematological malignancies: a randomized controlled trial. Clin Microbiol Infect 2021 1;27:69–75. 10.1016/j.cmi.2020.03.038 10.1016/j.cmi.2020.03.038 32272171

