# Correction to: Clinical impact and cost-consequence analysis of ePlex® blood culture identification panels for the rapid diagnosis of bloodstream infections: a single-center randomized controlled trial

**DOI:** 10.1007/s10096-024-04924-6

**Published:** 2024-08-29

**Authors:** Yvan Caspar, A. Deves, C. Richarme, M. Le Marechal, L. Ponderand, A.-L. Mounayar, S. Lejeune, J. Arata-Bardet, M. Gallouche, C. Recule, D. Maubon, C. Garnaud, M. Cornet, M. Veloso, B. Chabani, M. Maurin, S. David-Tchouda, P. Pavese

**Affiliations:** 1 Laboratoire de Bactériologie-Hygiène Hospitalière, CHU Grenoble Alpes, Grenoble, France; 2 Univ. Grenoble Alpes, CNRS, CHU Grenoble Alpes, CEA, IBS, Grenoble, 38000 France; 3 Service des Maladies infectieuses et tropicales, CHU Grenoble Alpes, Grenoble, France; 4 Univ. Grenoble Alpes, CNRS, UMR 5525, VetAgro Sup, Grenoble INP, CHU Grenoble Alpes, TIMC, Grenoble, 38000 France; 5 Service d’Hygiène Hospitalière, CHU Grenoble Alpes, Grenoble, France; 6 Laboratoire de Parasitologie-Mycologie, CHU Grenoble Alpes, Grenoble, France; 7 University Grenoble Alpes, CNRS, CHU Grenoble Alpes, TIMC, Grenoble, 38000 France; 8 Cellule d’ingénierie des données, CHU Grenoble Alpes, Grenoble, France; 9 Unité d’évaluation médico-économique, Pôle Santé Publique, CHU Grenoble Alpes, Grenoble, France; 10 CIC 1406 Grenoble, INSERM, Grenoble, 38000 France; 11 Univ. Grenoble Alpes, TIMC-Imag UMR 5525, Grenoble, 38000 France

**Correction to: European Journal of Clinical Microbiology & Infectious Diseases (2024) 43:1193–1203**

10.1007/s10096-024-04820-z

In the originally published article, the Abstract section was incorrectly captured without subheadings during typesetting.

The correct abstract is shown below:

**Abstract**

**Purpose** To assess clinical impact and perform cost-consequence analysis of the broadest multiplex PCR panels available for the rapid diagnosis of bloodstream infections (BSI).

**Methods** Single-center, randomized controlled trial conducted from June 2019 to February 2021 at a French University hospital with an institutional antimicrobial stewardship program. Primary endpoint was the percentage of patients with optimized antimicrobial treatment 12 h after transmission of positivity and Gram stain results from the first positive BC.

**Results** This percentage was significantly higher in the multiplex PCR (mPCR) group (90/105 = 85.7% %, CI95% [77.5 ; 91.8] vs. 68/107 = 63.6%, CI95% [53.7 ; 72.6]; *p* < 10^− 3^) at interim analysis, resulting in the early termination of the study after the inclusion of 309 patients. For patients not optimized at baseline, the median time to obtain an optimized therapy was much shorter in the mPCR group than in the control group (6.9 h, IQR [2.9; 17.8] vs. 26.4 h, IQR [3.4; 47.5]; *p* = 0.001). Early optimization of antibiotic therapy resulted in a non-statistically significant decrease in mortality from 12.4 to 8.8% (*p* = 0.306), with a trend towards a shorter median length of stay (18 vs. 20 days; *p* = 0.064) and a non-significant reduction in the average cost per patient of €3,065 (*p* = 0.15). mPCR identified all the bacteria present in 88% of the samples.

**Conclusion** Despite its higher laboratory cost, the use of multiplex PCR for BSI diagnosis leads to early-optimised therapy, seems cost-effective and could reduce mortality and length of stay. Their impact could probably be improved if implemented 24/7.

The original paper has been corrected.

