# Supplementary material for: Clinical impact and cost-consequence analysis of ePlex® blood culture identification panels for the rapid diagnosis of bloodstream infections: a single-center randomized controlled trial
Source: Eur J Clin Microbiol Infect Dis. 2024 Mar 27;43(6):1193–203. doi: 10.1007/s10096-024-04820-z (PMC11178566; doi:10.1007/s10096-024-04820-z)
Supplement: Supplementary file 1 — Supplementary Material 1 [file 10096_2024_4820_MOESM1_ESM.docx]

**SUPPLEMENTARY MATERIAL**

**Additional details about study design**

1518 patients were eligible from June 20^th^ 2019 to February 19^th^ 2021 but only 309 patients were enrolled and randomized. We did not fill a patient screening log during the study to have the number of patients screened and trace the reasons for screening failures. We did not intend to enroll all eligible patients because there were too many each weeks to allow their enrollment and follow-up according to the availability of the investigators and of clinical research assistants. Moreover, the COVID-19 pandemic slowed significantly patient’s enrollment and led to extend the study for a period of one year.

We apologize as the primary objective of the study was misspelled in the final version of the protocol and in ClinicalTrials.gov website (NCT03876990). Primary objective was not the delay but the percentage of success (optimized antibiotic treatment) at 12h after the validation of the positivity of the first blood culture and Gram stain result, which has been used to calculate sample size (see Statistical analysis paragraph in the Methods section). The delay between the validation of the positivity of the first blood culture and optimized treatment was however a secondary objective, reported in the Table 2.

Moreover some secondary outcomes initially included in the protocol were not analyzed because they were rare events: ICU admission or length of stay, antibiotic/antifungal treatment toxicity rate, recurrence of sepsis within 30 days, re-admission to hospital within 30 days.

**Assessment of effective and optimal intravenous antibiotic therapy, source of infection, and contamination**

Effective and optimal intravenous antibiotic therapy, source of infection, and contamination were assessed during the weekly multidisciplinary meeting that is part of local ASP for bacteremia.Optimized antimicrobial treatment was defined as optimal intravenous antimicrobial treatment according to the species identified, final AST profile, and current recommendations (considering optimal clinical efficacy, potential side effects and selection of bacterial resistance but not dosing). We did not have a checklist for adjudicating the main outcome. Bacteremia are too diverse to build a checklist applicable for all cases. The primary endpoint was determined according to French current treatment guidelines, depending on the source of infection:

- Practice guidelines for the management of adult community-acquired urinary tract infections ; DOI: [10.1016/j.medmal.2018.03.005](https://doi-org.insb.bib.cnrs.fr/10.1016/j.medmal.2018.03.005)
- Guidelines for management of intra-abdominal infections ; DOI: [10.1016/j.accpm.2015.03.005](https://doi-org.insb.bib.cnrs.fr/10.1016/j.accpm.2015.03.005)
- Antibiotic lock therapy for the conservative treatment of long-term intravenous catheter-related infections in adults and children: When and how to proceed? Guidelines for clinical practice 2020 ; [doi.org/10.1016/j.idnow.2021.02.004](https://doi.org/10.1016/j.idnow.2021.02.004)
- Pulmonary infections : <https://www.infectiologie.com/UserFiles/File/spilf/recos/2010-infvrb-spilf-afssaps.pdf>
- Others : Guidelines of the French Infectious Diseases Society : <https://www.infectiologie.com/fr/recommandations.html>

Most of the time, the antibiotic treatment considered as optimized treatment was the definitive intravenous treatment received by the patient for the bacteremia or fungemia episode, after adjustments of the antibiotic therapy based on the recommendation of the antibiotic stewardship team (Table S1), AST data and the primary source of infection identified. When intravenous treatment was not effective or not optimal but the first oral treatment was, the first oral treatment was considered optimized treatment (4% of the patients, see Table S1 below).

The assessors of the main outcome were the infectious disease physicians, clinical microbiologists and infection control specialists present each week during the weekly multidisciplinary meeting that is part of local ASP for bacteremia. These meetings included at least one infectious disease physician member of the ASP team, one clinical microbiologist and one infection control specialist but more may have been present at each meeting. Thus assessors changed regularly.

The main outcome was assessed once, during one of the weekly meetings, when all microbiological data were available (identification and AST results using standard of care method). If not all data were available, the patient was analysed during the next weekly meeting. For each patient included, the team determined if the pathogen identified was a contaminant or responsible of the bacteremia/fungemia episode and in the latter case, the source of infection. Then the team looked at the different antibiotic treatments received by the patient and determined which one was the optimal antimicrobial treatment according to French recommendations for the treatment of bacteremia and fungemia (considering optimal clinical efficacy, potential side effects and selection of bacterial resistance but not dosing) knowing the species identified and its final AST profile. During the study, the ASP team provided his advice daily as usual. The ASP consists of a mobile infectious disease team that reviews all positive BC results in real time, moving through medical units to see patients if necessary, providing audits and feedback on management, treatment, and infection control to the medical units until the final microbiological results are obtained. Institutional ASP remained unchanged throughout the study period. However, final decision of treatment choice always remained to the physician in charge of the corresponding patient.

The optimized treatment for the bacteremia/fungemia episode considered by the experts corresponded to :

- Final IV treatment for 255/301 (85%) of the patients.
- Absence or stop of any antibiotic treatment for 11/301 (4%) patients because the bacteria identified was a contaminant.
- First oral antibiotic treatment for 12/301 (4%) including 5 patients for which IV antibiotic treatment was ineffective or absent and 7 for which IV antibiotic treatment was not optimal (ecological impact because of too large antibiotic spectrum while not required [6] or potential selection of bacterial resistance [1])
- No optimized treatment possible because of early death for 1 patient
- IV antibiotic treatment but not the final IV one for 22/301 patients (7%) because other medical conditions lead to treatment modification or because final treatment decision of patient’s physician differed from ASP recommendation.

**Table S1 :** Optimized treatment categorization

| Optimized treatment | SoC group | mPCR group |
| --- | --- | --- |
| Final IV treatment | 134 | 121 |
| Absence or stop of any antibiotic treatment | 5 | 6 |
| First oral antibiotic treatment | 7 | 5 |
| IV antibiotic treatment but not the final IV one | 7 | 15 |

The experts concluded that the treatment was suboptimal when:

- the antibiotic spectrum was too large (ecological impact) while de-escalation was possible according to treatment guidelines
- an unnecessary antibiotic therapy had been used for contaminants (ecological impact)
- the treatment was ineffective because the bacteria was resistant to the antibiotic compound in vitro or because no antibiotic treatment had been started (initial clinical suspicion of a contaminant)
- the clinical efficacy of the treatment was suboptimal (e.g. : intermediate in-vitro susceptibility of the pathogen while low dosing was used)
- the treatment could result in the selection of resistant bacteria (e.g. : group 3 Enterobacterales treated with cefotaxime or ceftriaxone)
- potential side effects could occur (e.g. : vancomycin use while unnecessary)

Table below describes the assessment of each of these criteria in both study groups:

**Table S2:** Reasons for suboptimal treatment and categorization of patients with suboptimal treatment at T12h

| Criteria used for assigment as « suboptimal treatment » | | mPCR group  (n = 26) | SoC group (n = 60) | Additional Remark |
| --- | --- | --- | --- | --- |
| Too large antibiotic spectrum (ecological impact) | | 15 | 29 | In the mPCR group : 8 patients could have been optimized earlier based on mPCR result but adaptation was after 12h since T0 and 1 suboptimal treatment was consecutive to a mis-interpretation of mPCR result |
| Unnecessary antibiotic therapy for contaminants (ecological impact) | | 2 | 8 | In the mPCR group 1 patient could have been optimized earlier based on mPCR result but adaptation was after 12h since T0 |
| Ineffective treatment | Resistant in-vitro susceptibility of the pathogen | 7 | 3 | In the mPCR group 2 patients could have been optimized earlier based on mPCR result but adaptation was after 12h since T0 and 1 unoptimized treatment was consecutive to an invalid PCR result |
|  | No antibiotic therapy | 1 | 4 | In the mPCR group 1 patient could have been optimized earlier based on mPCR result but adaptation was after 12h since T0 |
| Suboptimal clinical efficacy | | 1 | 9 |  |
| Selection of bacterial resistance | | 0 | 3 |  |
| Too large antibiotic spectrum (ecological impact) AND Selection of bacterial resistance | | 0 | 1 |  |
| Too large antibiotic spectrum (ecological impact) AND potential side effects | | 0 | 1 |  |
| Unnecessary antibiotic therapy for contaminants (ecological impact) AND potential side effects | | 0 | 2 |  |

mPCR : multiplex PCR group ; SoC : Standard of care group

**Definition of antibiotic escalation, de-escalation and optimisation**

To classify the antibiotic spectrum as too large, we used the definition and the Tables proposed in the supplementary appendix of Banerjee and al (34) with the minor modifications or additions.

Antibiotic de-escalation was defined as changing to a narrower spectrum antibiotic (lower number category in Tables S3 and S4 below) or the cessation of one or more antibiotics. Antibiotic escalation was defined as changing to a broader spectrum antibiotic (higher number category in Tables S3 and S4 below) or addition of one or more antibiotics.Any other situation of modification of intravenous antibiotics was considered as optimisation. Single dose administration of aminiglycosides was not considered.

Classification of antibiotics with Gram-positive or Gram-negative activity is defined in Tables S3 and S4 below.

**Table S3**: Classification of Gram negative antibiotics

| Category | Spectrum | Antibiotics |
| --- | --- | --- |
| 1 | Narrowest | Ampicillin, cefazolin |
| 2 | Narrowest | Amoxicillin/clavulanic acid, 2^nd^ generation cephalosporins, 3^rd^ generation cephalosporins (except ceftazidime), PO fluoroquinolones (ciprofloxacin, levofloxacin, moxifloxacin), metronidazole, tetracyclines, trimethoprime, slufamethoxazole |
| 3 | Medium | Aminoglycosides (amikacin, gentamicin, tobramycin), ceftaroline, ceftazidime, IV fluoroquinolones ((ciprofloxacin, levofloxacin, moxifloxacin), fosfomycin |
| 4 | Medium broad | Antipseudomonal penicillin/penicilinase combinations, aztreonam, cefepim, ceftobiprole, ertapenem |
| 5 | Broadest | Anti-Pseudomonal carbapenems, ceftazidime/avibactam, ceftolozane/tazobactam, polymixins, tigecycline, cefiderocol |

**Table S4:** Classification of Gram positive antibiotics

| Category | Spectrum | Antibiotics |
| --- | --- | --- |
| 1 | Narrow | Ampicillin, azithromycin, cefazolin, clindamycin, erythromycin, oxacillin, trimethroprim-sulfamethoxazole, tetracycline |
| 2 | Medium | Dalbavancin, IV vancomycin |
| 3 | Broad | Ceftaroline, daptomycin, IV/PO linezolid, dalbavancin, quinupristin/dalfopristin, IV/PO tedizolid, ceftobiprole |

**Local epidemiology**

Local antibiotic resistance rates are low: 6-10% ESBL-producing isolates among *Enterobacterales* and 4-8% methicillin-resistant isolates among *S. aureus* BSI between 2016 and 2021

Figure S1: ePlex panels

| Gram-Positive panel | |
| --- | --- |
| *Bacillus cereus* group | |
| *Bacillus subtilis* group | |
| *Enterococcus* | |
| *Enterococcus faecalis* | |
| *Enterococcus faecium* | |
| *Staphylococcus* | |
| *Staphylococcus aureus* | |
| *Staphylococcus epidermidis* | |
| *Staphylococcus lugdunensis* | |
| *Streptococcus* | |
| *Streptococcus agalactiae* | |
| *Streptococcus anginosus* group | |
| *Streptococcus pneumoniae* | |
| *Streptococcus pyogenes* | |
| *Cutibacterium acnes* | |
| *Corynebacterium* | |
| *Lactobacillus* group | |
| *Listeria* | |
| *Listeria monocytogenes* | |
| *Micrococcus* | |
| Resistance genes | |
| *mecA* | *vanA* |
| *mecC* | *vanB* |
| Other targets | |
| **Pan Gram-Negative** | |
| **Pan *Candida*** | |

| Gram-Negative panel | |
| --- | --- |
| *Acinetobacter baumannii* | |
| *Bacteroides fragilis* | |
| *Citrobacter* | |
| *Cronobacter sakazakii* | |
| *Enterobacter* (non-*cloacae* complex) | |
| *Enterobacter cloacae* complex | |
| *Escherichia coli* | |
| *Fusobacterium nucleatum* | |
| *Fusobacterium necrophorum* | |
| *Haemophilus influenza* | |
| *Klebsiella oxytoca* | |
| *Klebsiella pneumoniae* group | |
| *Morganella morganii* | |
| *Neisseria meningitidis* | |
| *Proteus* | |
| *Proteus mirabilis* | |
| *Pseudomonas aeruginosa* | |
| *Salmonella* | |
| *Serratia* | |
| *Serratia marcescens* | |
| *Stenotrophomonas maltophilia* | |
| Resistance genes | |
| CTX-M | VIM |
| KPC | IMP |
| NDM | OXA |
| Other targets | |
| **Pan Gram-Positive** | |
| **Pan *Candida*** | |

| Fungal Pathogen Panel |
| --- |
| *Rhodotorula* |
| *Fusarium* |
| *Cryptococcus gattii* |
| *Cryptococcus neoformans* |
| *Candida lusitaniae* |
| *Candida dubliniensis* |
| *Candida famata* |
| *Candida kefyr* |
| *Candida guilliermondii* |
| *Candida tropicalis* |
| *Candida parapsilosis* |
| *Candida glabrata* |
| *Candida krusei* |
| *Candida albicans* |
| *Candida auris* |

Table S5**:** Treatment duration for the main intravenous anti-infectious agents used for BSI treatment.

| Antibiotic | During the first 4 days after Gram stain result (h) | | |
| --- | --- | --- | --- |
|  | SoC | mPCR | p-value^1^ |
| Piperacillin/tazobactam |  |  |  |
| Number of patients | 62 | 56 |  |
| med [IQR] | 51.8 [25.3 ; 96] | 36.2 [8.7 ; 91.3] | 0.118 |
| Daptomycin |  |  |  |
| Number of patients | 28 | 25 |  |
| med [IQR] | 48.6 [27.7 ;89.8] | 40.4 [14.5 ;80.4] | 0.236 |
| Ceftriaxone |  |  |  |
| Number of patients | 30 | 30 |  |
| med [IQR] | 39.4 [28.5 ; 69.2] | 69.8 [26 ; 87.3] | 0.160 |
| Cefotaxime |  |  |  |
| Number of patients | 24 | 31 |  |
| med [IQR] | 32.8 [19.3 ; 63.9] | 47 [18.2 ; 74] | 0.671 |
| Carbapenems |  |  |  |
| Number of patients | 8 | 20 |  |
| med [IQR] | 68.8 [45.3;84.7] | 66.9 [26.8;92.9] | 0.838 |
| Metronidazole |  |  |  |
| Number of patients | 19 | 22 |  |
| med [IQR] | 49.2 [32.2;90] | 58.1 [31.8;95.5] | 0.763 |
| Cefepim |  |  |  |
| Number of patients | 22 | 24 |  |
| med [IQR] | 70 [39.5;89.3] | 67.4 [15.6;88.4] | 0.361 |
| Cefazolin |  |  |  |
| Number of patients | 20 | 19 |  |
| med [IQR] | 70.6 [54.1;88.4] | 72.6 [29.5;91.7] | 0.673 |

*Abbreviations: SoC, standard of care; mPCR: multiplex polymerase chain reaction; IQR, interquartile range*

*^1^: Mann-Whitney U test*

Table S6: Gram stain result of positive BC

| Gram stain result | SoC group  (n=153) | mPCR group  (n=148) |
| --- | --- | --- |
| GNB  GPC staphylococci  GPC streptococci  Yeasts  GPB  Polymicrobial | 79 (51.6%)  41 (26.8%)  19 (12.4%)  6 (3.9%)  3 (2%)  5 (3.3%) | 75 (50.6%)  39 (26.4%)  22 (14.9%)  3 (2%)  2 (1.4%)  7 (4.7%) |

*Abbreviations: SoC, standard of care; mPCR: multiplex polymerase chain reaction; GNB : Gram-negative bacteria; GPC : Gram-positive cocci*

Table S7: Description of bacterial species in monomicrobial and polymicrobial BSI in the SoC and mPCR groups.

| Bacterial species | SoC | | mPCR | |
| --- | --- | --- | --- | --- |
|  | n | % | n | % |
| *E. coli* | 37 | 24.2 | 36 | 24.3 |
| *S. epidermidis* | 15 | 9.8 | 14 | 9.5 |
| *S. aureus* | 10 | 6.5 | 13 | 8.8 |
| *E. cloacae* | 8 | 5.2 | 6 | 4.1 |
| *E. faecalis* | 4 | 2.6 | 8 | 5.4 |
| *K. pneumoniae* | 5 | 3.3 | 7 | 4.7 |
| *P. mirabilis* | 3 | 2.0 | 4 | 2.7 |
| *S. haemolyticus* | 3 | 2.0 | 4 | 2.7 |
| *P. aeruginosa* | 3 | 2.0 | 3 | 2.0 |
| *S. anginosus group* | 3 | 2.0 | 3 | 2.0 |
| *S. agalactiae* | 2 | 1.3 | 3 | 2.0 |
| *C. koseri* | 1 | 0.7 | 3 | 2.0 |
| *E. aerogenes* | 2 | 1.3 | 1 | 0.7 |
| *K. pneumoniae + E. cloacae* | 1 | 0.7 | 2 | 1.4 |
| *M. luteus* | 2 | 1.3 | 1 | 0.7 |
| *S. marcescens* | 2 | 1.3 | 1 | 0.7 |
| *S. aureus + S. epidermidis* | 2 | 1.3 | 1 | 0.7 |
| *S. epidermidis + S. haemolyticus* | 2 | 1.3 | 1 | 0.7 |
| *S. mitis/oralis* | 2 | 1.3 | 1 | 0.7 |
| *S. pyogenes* | 2 | 1.3 | 1 | 0.7 |
| *C. acnes* | 2 | 1.3 | 0 | 0.0 |
| *C. albicans* | 1 | 0.7 | 1 | 0.7 |
| *C. dubliniensis* | 1 | 0.7 | 1 | 0.7 |
| *C. freundii* | 1 | 0.7 | 1 | 0.7 |
| *E. faecium* | 2 | 1.3 | 0 | 0.0 |
| *K. oxytoca* | 0 | 0.0 | 2 | 1.4 |
| *M. morganii* | 2 | 1.3 | 0 | 0.0 |
| *Salmonella sp* | 1 | 0.7 | 1 | 0.7 |
| *S. hominis* | 1 | 0.7 | 1 | 0.7 |
| *S. maltophilia* | 1 | 0.7 | 1 | 0.7 |
| *S. dysgalacatiae* | 0 | 0.0 | 2 | 1.4 |
| *S. pneumoniae* | 1 | 0.7 | 1 | 0.7 |
| *Acinetobacter pittii* | 1 | 0.7 | 1 | 0.7 |
| *Acinetobacter calcoaceticus* | 1 | 0.7 | 0 | 0.0 |
| *A. baumanii* | 0 | 0.0 | 1 | 0.7 |
| *Bacteroides thetaiotaomicron* | 1 | 0.7 | 0 | 0.0 |
| *Bacteroides vulgatus + Bacteroides caccae* | 1 | 0.7 | 0 | 0.0 |
| *B. cereus* group | 0 | 0.0 | 1 | 0.7 |
| *C. glabrata* | 1 | 0.7 | 0 | 0.0 |
| *C. kefyr + C. krusei + C. tropicalis* | 1 | 0.7 | 0 | 0.0 |
| *C. krusei* | 0 | 0.0 | 1 | 0.7 |
| *C. lusitaniae* | 1 | 0.7 | 0 | 0.0 |
| *C. tropicalis* | 1 | 0.7 | 0 | 0.0 |
| *E. faecium + Clostridium cadaveris* | 1 | 0.7 | 0 | 0.0 |
| *E. faecium+ E. coli + K. pneumoniae* | 0 | 0.0 | 1 | 0.7 |
| *E. faecalis +M. morganii* | 0 | 0.0 | 1 | 0.7 |
| *E. faecalis + M. morganii + Hafnia alvei* | 1 | 0.7 | 0 | 0.0 |
| *E. coli + K. pneumoniae* | 1 | 0.7 | 0 | 0.0 |
| *E. coli + P. mirabilis* | 0 | 0.0 | 1 | 0.7 |
| *Gemella haemolysans* | 0 | 0.0 | 1 | 0.7 |
| *Haemophilus parainfluenzae* | 0 | 0.0 | 1 | 0.7 |
| *Klebsiella planticola* | 1 | 0.7 | 0 | 0.0 |
| *K. oxytoca + E. cloacae + C. freundii + Cronobacter sp + Acinetobacter berezinae + Raoultella ornithinolytica* | 0 | 0.0 | 1 | 0.7 |
| *K. pneumoniae + E. cloacae + A. pittii* | 1 | 0.7 | 0 | 0.0 |
| *K. pneumoniae + S. gallolyticus* | 0 | 0.0 | 1 | 0.7 |
| *Lactobacillus paracasei* | 1 | 0.7 | 0 | 0.0 |
| *Leptotrichia spp.* | 1 | 0.7 | 0 | 0.0 |
| *Moraxella nonliquefaciens + F. nucleatum* | 1 | 0.7 | 0 | 0.0 |
| *Moraxella osloensis* | 1 | 0.7 | 0 | 0.0 |
| *M. luteus + M. osloensis* | 0 | 0.0 | 1 | 0.7 |
| *M. morganii + P. mirabilis* | 1 | 0.7 | 0 | 0.0 |
| *Pantoea agglomerans + P. putida* | 1 | 0.7 | 0 | 0.0 |
| *P. agglomerans* | 1 | 0.7 | 0 | 0.0 |
| *Parvimonas micra* | 1 | 0.7 | 1 | 0.7 |
| *Peptoniphilus indolicus* | 0 | 0.0 | 1 | 0.7 |
| *P. vulgaris* | 0 | 0.0 | 1 | 0.7 |
| *R. ornithinolytica* | 0 | 0.0 | 1 | 0.7 |
| *S. capitis* | 2 | 0.7 | 0 | 0.0 |
| *S. gallolyticus* | 0 | 0.0 | 1 | 0.7 |
| *S. pettenkoferi* | 1 | 0.7 | 0 | 0.0 |
| *Staphylococcus equorum* | 1 | 0.7 | 0 | 0.0 |
| *Streptococcus mutans* | 0 | 0.0 | 1 | 0.7 |
| *S. aureus + E. coli* | 1 | 0.7 | 0 | 0.0 |
| *S. epidermidis + E. cloacae* | 1 | 0.7 | 0 | 0.0 |
| *S. epidermidis + S. capitis* | 0 | 0.0 | 1 | 0.7 |
| *S. epidermidis + S. hominis* | 1 | 0.7 | 0 | 0.0 |
| *S. epidermidis +S. hominis + E. cloacae* | 0 | 0.0 | 1 | 0.7 |
| *S. epidermidis + S. hominis + S. capitis* | 1 | 0.7 | 0 | 0.0 |
| *S. epidermidis + S. hominis + S. haemolyticus* | 1 | 0.7 | 0 | 0.0 |
| *S. haemolyticus + E. coli* | 1 | 0.7 | 0 | 0.0 |
| *S. hominis + S. haemolyticus + S. capitis* | 0 | 0.0 | 1 | 0.7 |
| *S. maltophilia + A. ursingii* | 0 | 0.0 | 1 | 0.7 |
| *S. anginosus group + B. fragilis* | 0 | 0.0 | 1 | 0.7 |
| *S. mitis/oralis + E. coli* | 0 | 0.0 | 1 | 0.7 |

*Abbreviations: SoC, standard of care; mPCR: multiplex polymerase chain reaction*

Table S8: Resistance mechanisms detected by SoC in both groups

|  | SoC group  (n=153) | mPCR group  (n=148) |
| --- | --- | --- |
| Resistance mechanism | Number of resistant isolates | Number of resistant isolates |
| - Methicillin resistance | 19 | 17 |
| - ESBL production | 5 | 6 |
| - Hyperproduction of cephalosporinase | 2 | 2 |

*Abbreviations: SoC, standard of care; mPCR: multiplex polymerase chain reaction; ESBL : extended spectrum beta-lactamase*

Table S9: Positive percent agreement (PPA) and negative percent agreement (NPA) of the ePlex^®^ BCID-GN Panel

| BCID-GN Panel Targets | Identification and resistance results by SoC testing | mPCR PPA (%) | mPCR NPA (%) |
| --- | --- | --- | --- |
| *Escherichia coli* | *E. coli* | 37/38 (97.4) | 43/43 (100) |
| *Klebsiella pneumonia* group | *K. pneumoniae* | 11/11 (100) | 70/70 (100) |
| *Klebsiella oxytoca* | *K. oxytoca* | 3/3 (100) | 78/78 (100) |
| *Proteus mirabilis* | *P. mirabilis* | 5/5 (100) | 76/76 (100) |
| *Proteus* | 5 *P. mirabilis,* 1 *Proteus vulgaris* | 6/6 (100) | 75/75 (100) |
| *Enterobacter cloacae* complex | *E. cloacae* complex | 9/9 (100) | 72/72 (100) |
| *Enterobacter* (non-*cloacae* complex) | K. aerogenes | 1/1 (100) | 80/80 (100) |
| *Citrobacter sp* | 3 *C. koseri,* 2 *C. freundii* | 4/5 (80) | 76/76 (100) |
| *Serratia marcescens* | *S. marcescens* | 1/1 (100) | 80/80 (100) |
| *Serratia* | No other species | 1/1 (100) | 80/80 (100) |
| *Morganella morganii* | *M. morganii* | 1/1 (100) | 80/80 (100) |
| *Cronobacter sakazakii* | None |  | 81/81 (100) |
| *Salmonella sp* | *S. enterica* | 1/1 (100) | 80/80 (100) |
| *Pseudomonas aeruginosa* | *P. aeruginosa* | 3/3 (100%) | 78/78 (100%) |
| *Acinetobacter baumanii* | *A. baumanii* | 1/1 (100) | 80/80 (100) |
| *Stenotrophomonas maltophilia* | *S. maltophilia* | 2/2 (100%) | 79/79 (100%) |
| *Haemophilus influenzae* | None |  | 81/81 (100) |
| *Neisseria meningitidis* | None |  | 81/81 (100) |
| *Bacteroides fragilis* | *B. fragilis* | 1/1 (100) | 80/80 (100) |
| *Fusobacterium nucleatum* | None |  | 81/81 (100) |
| *Fusobacterium necrophorum* | None |  | 81/81 (100) |
| Pan *Candida* | None |  | 81/81 (100) |
| Pan Gram-Positive | *Streptococcus anginosus* group (1); *S. gallolyticus* (1), *S. mitis/oralis* (1), *E. faecium* (1); *E. faecalis* (1), *S. epidermidis* + *S. hominis* (1) | 5/6 (83) | 75/75 (100) |
| *bla_CTX-M_* gene | ESBL-producing *: E. coli* (2); *K. pneumoniae* (1); *E. cloacae* (1) | 4/5 (100) | 71/71 (100) |
| *bla_OXA_, bla_VIM_, bla_NDM_, bla_KPC_, bla_IMP_* | None |  | 74/74 (100) |
| No Targets Detected (of-panel) | *Acinetobacter berezinae* + *Raoultella ornithinolytica* (1); *Acinetobacter pittii* (1); *Acinetobacter ursingii* (1); *Haemophilus parainfluenzae* (1); *Raoultella ornithinolytica* (1); *Moraxella osloensis* (1) |  |  |

*Abbreviations: SoC, standard of care; mPCR: multiplex polymerase chain reaction; ESBL : extended spectrum beta-lactamase, 2 BCID GN cartridges were invalid (see Table S9)*

Table S10: Positive percent agreement (PPA) and negative percent agreement (NPA) of the ePlex^®^ BCID-GP Panel

| BCID-GP Panel Targets | Identification and resistance results by SoC testing | mPCR PPA (%) | mPCR NPA (%) |
| --- | --- | --- | --- |
| *Staphylococcus aureus* | *S. aureus* | 14/14 (100) | 53/53 (100) |
| *Staphylococcus epidermidis* | *S. epidermidis* | 15/18 (83) | 49/49 (100) |
| *Staphylococcus lugdunensis* | *None* |  | 67/67 (100) |
| *Staphylococcus* | *S. hominis* (2); *S. capitis* (1); *S. haemolyticus* (5) ; *S. hominis* + *S. haemolyticus* + *S. capitis* (1) | 35/36 (97) | 30/30 (100) |
| *Enterococcus faecalis* | *E. faecalis* | 9/9 (100) | 58/58 (100) |
| *Enterococcus faecium* | *E. faecium* | 1/1 (100) | 66/66 (100) |
| *Enterococcus* | None other species | 10/10 (100) | 57/57 (100) |
| *Streptococcus pneumoniae* | None |  | 67/67 (100) |
| *Streptococcus pyogenes* | *S. pyogenes* | 1/1 (100) | 66/66 (100) |
| *Streptococcus agalactiae* | *S. agalactiae* | 3/3 (100) | 64/64 (100) |
| *Streptococcus anginosus* group | *S. milleri* group | 3/3 (100) | 64/64 (100) |
| *Streptococcus* | *S. mitis/oralis* group (2); *S. gallolyticus* (2); *S. dysgalactiae* (2); *S. mutans* (1); | 14/14 (100) | 53/53 (100) |
| *Corynebacterium* | None | 1/1 (100) | 66/66 (100) |
| *Micrococcus* | *M. luteus* | 2/2 (100) | 65/65 (100) |
| *Lactobacillus* | None |  | 67/67 (100) |
| *Bacillus cereus* group | *B. cereus* | 1/1 (100) | 66/66 (100) |
| *Bacillus subtilis* group | None |  | 67/67 (100) |
| *Cutibacterium acnes* | None |  | 67/67 (100) |
| Pan Gram-Negative | *M. morganii* (1); *K; pneumoniae* (2); *B. fragilis* (1); *E. coli* (2); *M. osloensis* (1); *E. cloacae* complex (1) | 7/7 (100) | 60/60 (100) |
| Pan *Candida* | None |  | 67/67 (100) |
| *mecA* gene | Methicillin-resistant *: S. aureus* (1); *S. epidermidis* (8); *S. haemolyticus* (4); *S.epidermidis* + *S. capitis* (1); *S. epidermidis* + *S. haemolyticus* (1); *S. epidermidis* + *S. hominis* (1); *S.hominis* + *S. haemolyticu*s + *S. capitis* (1) | 16/17 (94) | 20/20 (100) |
| *vanA* gene | None |  | 10/10 (100) |
| *vanB* gene | None |  | 10/10 (100) |
| No Targets Detected (off-panel) | *Gemella haemolysans* (1); *Parvimonas micra* (1); *Peptoniphilus indolicus* (1); |  |  |

*Abbreviations: SoC, standard of care; mPCR: multiplex polymerase chain reaction*

*2 BCID GP cartridges were invalid (see Table 4)*

Table S11: Positive percent agreement (PPA) and negative percent agreement (NPA) of the ePlex^®^ BCID-FP Panel

| BCID-FP Panel Targets | Identification results by SoC testing | mPCR PPA (%) | mPCR NPA (%) |
| --- | --- | --- | --- |
| *Candida albicans* | *C. albicans* | 1/1 (100) | 2/2 (100) |
| *Candida dubliniensis* | *C. dubliniensis* | 1/1 (100) | 2/2 (100) |
| *Candida krusei* | *C. krusei* | 1/1 (100) | 2/2 (100) |
| Other targets | None (*Candida auris, Candida glabrata, Candida kefyr, Candida guilliermondii, Candida tropicalis, Candida parapsilosis, Candida famata, Candida lusitaniae, Candida tropicalis, Cryptococcus gattii, Cryptococcus neoformans, Fusarium, Rhodotorula*) |  | 3/3 (100) |

*Abbreviations: SoC, standard of care; mPCR: multiplex polymerase chain reaction*

Table S12**:** Performance of the ePlex^®^ BCID Panels for polymicrobial BSI

| Bacteria/Yeast species identified  (SoC result) | Number of samples | Targets identified by mPCR |
| --- | --- | --- |
| *E. cloacae* complex *+ K. pneumoniae* | 2 | *E. cloacae* complex*; K. pneumonia group* |
| *E. coli + P. mirabilis* | 1 | *E. coli; P. mirabilis* |
| *S. aureus + S. epidermidis* | 1 | *S. aureus; S. epidermidis; Staphylococcus sp.* |
| *S. epidermidis+ S. haemolyticus* | 1 | *Staphylococcus sp.* |
| *S. epidermidis + S. capitis* | 1 | *Staphylococcus sp., S. epidermidis* |
| *E. faecalis + M. morganii* | 1 | *E. faecalis; Pan-GN; M. morganii; Pan-GP* |
| *S. gallolyticus + K. pneumoniae* | 1 | *K. pneumoniae; Pan-GP; Streptococcus sp; Pan-GN* |
| *S. anginosus group + B. fragilis* | 1 | *S. anginosus group; Pan-GN; B. fragilis; Pan-GP* |
| *S. mitis/oralis + E. coli* | 1 | *E. coli; Pan-GP Streptococcus sp.; Pan-GN* |
| *E. faecium + E. coli+ K. pneumoniae* | 1 | *E. faecium; Pan-GN; E. coli; K. pneumoniae group* |
| *M. luteus+ M. osloensis×* | 1 | *Micrococcus sp; Pan-GN* |
| *S. maltophilia +A. ursingii×* | 1 | *S. maltophilia* |
| *S. hominis + S. haemolyticus + S. capitis* | 1 | *Staphylococcus sp* |
| *E. cloacae* complex *+ S. epidermidis + S. hominis* | 1 | *E. cloacae* complex*; Pan-GP; Staphylococcus sp.; Pan-GN* |
| *C. freundii + E. cloacae* complex *+ K. oxytoca + Cronobacter sp + Acinetobacter berezinae× + Raoultella ornithinolytica×* | 1 | *Citrobacter sp; E. cloacae* complex*; K. oxytoca;* |
| Total | 16 | Identification of all bacteria : 11/16 samples |

*Abbreviations: SoC, standard of care; mPCR: multiplex polymerase chain reaction*

*×off-panel target*

*Pan GP: Pan Gram-Positive target (detected in the BCID-GN Panel)*

*Pan GN: Pan Gram-Negative target (detected in the BCID-GP Panel)*

Table S13: Discrepancies between mPCR and SoC results

| N° | SoC result | mPCR result | Undetected target | Hypothesis/Reason for failure |
| --- | --- | --- | --- | --- |
| 1 | *S. anginosus group* | Invalid |  | Cartridge failure/PCR inhibitors |
| 2 | *S. pneumoniae* | Invalid |  |  |
| 3 | ESBL-producing *E. cloacae* | Invalid |  |  |
| 4 | *E. coli* | Invalid |  |  |
| 5 | MR *S. epidermidis* | No target detected | *Staphylococcus* sp.; *S. epidermidis*; *mecA* | Lack of sensitivity or variant not detected by mPCR primers |
| 6 | *C. freundii* | No target detected | *Citrobacter* sp. | Lack of sensitivity or variant not detected by mPCR primers |
| 7 | *E. coli* | No target detected | *E. coli* | Lack of sensitivity or variant not detected by mPCR primers |
| 8 | ESBL-producing *E. coli* | *E. coli* | *bla_CTX-M_* | Lack of sensitivity at first try, *bla_CTX-M_* gene detected when retested in saline solution |
| 9 | MR *S. epidermidis*  *+* MR *S. haemolyticus* | *Staphylococcus* sp., *mecA* | *S. epidermidis* | Lack of sensitivity or variant not detected by mPCR primers |
| 10 | MR *S. hominis*  *+* MR *S. epidermidis*  *+ E. cloacae complex* | *E. cloacae* complex  *Staphylococcus* sp, *mecA* | *S. epidermidis* | Lack of sensitivity or variant not detected by mPCR primers |
| 11 | *E. faecium + E. coli+ K. pneumoniae* | *E. faecium*; Pan-GN; *E. coli*; *K. pneumoniae* | Pan-GP | Lack of sensitivity or variant not detected by mPCR primers |

*Abbreviations: SoC: standard of care; mPCR: multiplex polymerase chain reaction; MS: methicillin-susceptible; MR: methicillin-resistant; ESBL : extended spectrum beta-lactamase*

*Pan GP: Pan Gram-Positive target (detected in the BCID-GN Panel)*

*Pan GN: Pan Gram-Negative target (detected in the BCID-GP Panel)*
